# The Metropolitan Hospital Saturday Fund

**Published:** 1891-01-31

**Authors:** 


### The Metropolitan Hospital Saturday Fund.

Under the presidency of Mr. Reginald Acland, the chairman of the Fund, a largely attended meeting of the delegates of the Hospital Saturday Fund was held at the offices, Farringdon Road, last Saturday evening, for the purpose of considering a report relating to the office staff, which had been drawn up by a Special Committee after some nine sittings. The meeting, which lasted for five hours, proved to be a very excited one. The report of the Committee, which was moved by Mr. J. G. Lemon, stated the present staff and the salaries paid to be as follows: Mr. Frewer (General Secretary), £250; Mr. Bunn (Organising Secretary), £250; Mr. Webster, £200; Mr. Taylor, £200; Mr. Jones, £150; Mr. White, £110; Mr. Jolly, £90; Mr. Green, £54; Mr. Temple, £65; and a boy, £26. The Committee stated that they had come to the conclusion that what was most required was some one efficient person as secretary, whose duty it would be to direct and be responsible to the Committee for the whole of the work. The work of the fund had been seriously hampered by the present system of divided authority, and it was essential that there should be in future only one secretary. They recommended, therefore, that with the view to the appointment of one secretary, the engagements of Mr. Frewer and Mr. Bunn should be determined by three months' notice, and that applications from them should be considered with others. After recommending the termination of the engagement of the two other officials, the Committee added that they had considered also what staff was required to carry on the work of Fund. They recommended that the office staff should be as follows:—A secretary, £250; a cash and ledger clerk, £120; a clerk specially to attend to the indoor work of the organising department, £120; a general clerk, £75; a porter, £65; and a boy, £26. This would effect a saving of £300 per year, even if all the assistant organising secretaries were retained. The Committee, however, being of opinion that there was, at least for another year, sufficient work to be done outside the office to occupy the time of the three organising secretaries, recommended that they should be continued for another year at their present salaries. The adoption of the report having been seconded by Mr. Compton, a long and acrimonious discussion followed, in the course of which charges of a personal nature were freely made. Towards the close of the proceedings Mr. Hutchinson moved as an amendment that so much of the report as referred to the question of dual control should be adjourned for six months, and that in the meantime the secretaries should be furnished with sufficient clerical assistance. On a division being taken, this was carried by 34 votes to 26. The result was declared amid a scene of considerable excitement, which was increased when the Chairman and several delegates intimated their intention of resigning. The meeting then separated, in considerable disorder.

